# A Tribute to Professor Jianguo Wu

**DOI:** 10.3390/v15081720

**Published:** 2023-08-11

**Authors:** Xin Chen

**Affiliations:** Guangdong Provincial Key Laboratory of Virology, Key Laboratory of Viral Pathogenesis & Infection Prevention and Control, Institute of Medical Microbiology, Jinan University, Guangzhou 510632, China; chenx@jnu.edu.cn

It has been a couple of months since Professor Jianguo Wu left us. Whenever I think about Professor Wu, his familiar voice and kind smile always appear in front of me. As a famous virologist, the former Director of the National Key Laboratory of Virology, Wuhan University and a Professor/Dean of Guangdong Provincial Key Laboratory of Virology, Institute of Medical Microbiology, JINAN University, Professor Wu dedicated himself to virology research in China. He was a refined and engaging teacher, a charismatic professor, a gentle man and scholar. We deeply miss him for his wise words, encouragement, patient guidance and contagious singing.

It is a great fortune to have been taught and guided by Professor Wu throughout my academic life. I first met Professor Wu in 2006, when I was beginning my master’s study at Wuhan University, China. One of my first memories of Professor Wu was in the first supervision day, he asked me about my research interests in virology fields and future dreams. During our discussion, Professor Wu impressed me as a virologist with contagious enthusiasm and passion. Professor Wu had a strong faith that the future of students can be changed through patient education and kind guidance. As a supervisor, he encouraged and motivated you through the research. He always kept you on track to what was most novel and impactful among your studies. He was very generous to each student and provided us with various opportunities to attend academic conferences, summer schools and overseas studies. These academic activities and seminar training not only expanded our horizons, but also provided us with a diverse scope in fundamental and translational research, inspiring us in future research.

As a professor, he was always energetic and willing to embrace new challenges. He was open-minded to new technologies and never set limits on himself. In the beginning of Professor Wu’s research, by employing molecular biotechniques, he characterized DNA-dependent RNA polymerase and studied mechanisms regulating cell division and cell cycles from a tiny freshwater bacterium, *Caulobacter* [1,2,3]. After Professor Wu set up a lab at Wuhan University, China, he put extensive interests into human medical virology. Professor Wu and his group made fundamental progress in the mechanism of diverse viral infection, replication and pathogenesis, covering most common viruses [4,5,6,7,8,9,10,11,12,13,14,15,16] and viral coinfections [17,18,19,20] in humans. In addition, Professor Wu devoted himself to mechanism research of viral-induced oncogenesis and exploring virus-based cancer therapeutic strategies. His group explored regulatory mechanisms for Hepatitis B virus (HBV) and Hepatitis C virus (HCV)-driven liver tumorigenesis [21,22,23,24,25,26,27,28], and identified a novel HBx-interacting protein, YueF, functioning as a tumor suppressor [22]. One of Professor Wu’s greatest interests was to understand the regulatory mechanisms of host immunity and viral infections [29,30,31,32,33,34,35,36,37,38,39,40,41]. He and his collaborators identified multiple pathways in the regulation of inflammatory factors during viral infection [42,43,44,45,46]. His group also made impressive scientific discoveries in regulatory mechanisms of NLRP3 inflammasome activation during viral infection [47,48,49,50,51,52]. Moreover, Professor Wu always kept an open mind to new technologies and methodologies to develop antiviral agents and vaccines. He and his collaborators explored single-chain intracellular antibodies [53], natural medicinal compounds [54,55,56], nanomaterials [57], single-cell based siRNA screens [58,59,60] and vaccines [61,62] to inhibit or prevent viral replication. His vision and scientific attitude had a great influence on our future studies and career plans. Professor Wu proved to us that lifelong learning is a worthwhile process. 

As a founding member, Professor Wu was appointed the Dean of the Institute of Medical Microbiology at JINAN University. He still kept a great enthusiasm to guide undergraduate and postgraduate students. He was happy to provide every bit of guidance and share his expertise in virology research. As a mentor, Professor Wu encouraged, motivated and supported you to become a better researcher in the scientific world. He helped his colleagues build goals and assisted them in focusing on their goals. He continuously gave you practical advice and shared knowledge to improve your professional and personal development. Meanwhile, Professor Wu was a patient listener who was willing to listen to your problems. He always offered you constructive feedback and guided you through your problems. With the support and guidance of Professor Wu, many of his students have gone on to have distinguished careers in scientific research fields, biomedical industries, public service and other fields.

Though I was his former master’s student and joined his lab at JINAN University for a brief time, I feel honored to have had the precious opportunity to learn from him. I was deeply influenced by him not just by his professional attitude, but by his charm and personality. Professor Wu was a well-known gentleman and scholar for his erudition, modesty, graciousness and generosity. Despite the many successes Professor Wu had achieved, he remained humble. In his work life, Professor Wu always kept great optimism among all the difficulties. He kept encouraging us that optimism brings hope. His positive life attitude greatly influenced the people that surrounded him. For the students who were struggling, he was always ready to lend a helping hand and willing to share his knowledge and wisdom. Whenever I want to give up on my study or work, his warm encouragement and determined face always reminds me to keep on going. What impressed us most was that Professor Wu was merciful man with a generous heart. During the COVID-19 outbreak, his group donated tons of disinfectant to universities and hospitals to support the protective measures. His charm and personality had enormous positive impacts on his students and colleagues. Under the influence of the holy spirit, more and more students are willing to engage in charitable activities and civic duties. We will always remember Professor Wu for the time we spent together.
